# Localized surface plasmon resonance-based point-of-care testing for pediatric sepsis

**DOI:** 10.3389/fped.2026.1880296

**Published:** 2026-07-16

**Authors:** Xiao Zhang, Jing Zhang, Minghao Wang, Zelin Liu, Zhibo Gai, Xia Li

**Affiliations:** 1 Department of Pediatrics, Liaocheng People’s Hospital, Liaocheng, Shandong, China; 2 Fengtai Rehabilitation Hospital of Beijing Municipality (Tieying Hospital), Beijing, China; 3 School of Medicine, Shandong University of Traditional Chinese Medicine, Ji’nan, Shandong, China; 4 Experimental Center, Shandong University of Traditional Chinese Medicine, Jinan, China; 5 Key Laboratory of Traditional Chinese Medicine Classical Theory, Ministry of Education, Shandong University of Traditional Chinese Medicine, Jinan, China

**Keywords:** biomarkers, critical care, early diagnosis, localized surface plasmon resonance (LSPR), pediatric sepsis, point-of-care testing (POCT)

## Abstract

Sepsis is a leading cause of child mortality worldwide, yet conventional diagnostic methods are too slow for time-critical management. This review examines the emerging role of localized surface plasmon resonance (LSPR) technology in developing point-of-care testing (POCT) platforms for pediatric sepsis. We outline the clinical need for rapid, accurate diagnostics, then explain the physical principles of LSPR that enable label-free, sensitive detection of biomolecular interactions. LSPR-based POCT systems for sepsis are critically analyzed, including their design, target biomarkers, and performance metrics from validation studies. The potential clinical impact is evaluated in both high-resource and limited-resource settings. Finally, future directions, technological innovations, regulatory barriers, and research gaps are discussed. The evidence suggests that LSPR-POCT offers a paradigm shift, providing the speed and sensitivity needed to effectively address the diagnostic challenges of pediatric sepsis.

## 1 Introduction

Sepsis, defined as life-threatening organ dysfunction resulting from a dysregulated host response to infection, poses a significant and persistent threat to global child health (1). It remains a leading cause of death in infants and children, with outcomes heavily dependent on the timeliness of recognition and the rigor of initial management (1). The challenge is particularly acute in pediatric populations owing to the nonspecific and variable clinical presentation of sepsis in children, which often mimics other, less severe conditions and leads to diagnostic uncertainty (2). This ambiguity is compounded by a historical reliance on diagnostic definitions and clinical guidelines extrapolated from adult studies—tools that, while clinically useful, lack formal validation in the heterogeneous pediatric population (3). Consequently, the window for effective therapeutic intervention, often measured in minutes to hours, is frequently missed, resulting in high rates of morbidity, mortality, and long-term sequelae (4).

The limitations of conventional diagnostic paradigms, which rely heavily on central laboratory facilities for culture-based pathogen identification and biomarker quantification, are a primary contributor to these poor outcomes. Such methods are inherently slow, often requiring 24 to 72 h to yield definitive results, thereby delaying targeted antimicrobial therapy and appropriate supportive care (5). In response to this critical unmet need, there has been a significant push toward the development and implementation of point-of-care testing (POCT) in pediatric healthcare settings. POCT offers the promise of rapid, on-site biochemical and microbiological evaluation, bypassing the delays associated with central laboratories and enabling swift clinical decision-making (6). Among emerging POCT technologies, those based on localized surface plasmon resonance (LSPR) have garnered considerable attention for their potential to deliver ultrasensitive, label-free, and multiplexed biomarker detection in a format suitable for bedside use (7).

This literature review aims to provide a comprehensive and in-depth analysis of the application of LSPR-based POCT for the diagnosis of pediatric sepsis. It synthesizes findings from a wide range of studies to construct a detailed picture of the clinical necessity, underlying technological principles, current state of system development, potential for clinical integration, and future trajectory of this promising field. By critically evaluating the existing evidence, this review seeks to elucidate how LSPR technology can address the specific diagnostic challenges of pediatric sepsis and ultimately contribute to improved clinical outcomes for this vulnerable patient population. The scope encompasses the entire translational pipeline, from the fundamental physics of LSPR to the practical considerations of clinical implementation in diverse healthcare environments.

## 2 Clinical imperatives for pediatric sepsis diagnosis

### 2.1 Epidemiology and prognostic challenges

Pediatric sepsis remains a leading cause of global childhood mortality, accounting for millions of deaths annually, with mortality rates reaching 50%–60% among critically ill children in the pediatric intensive care unit (PICU) (1, 8). Epidemiological data from Brazil (SPREAD-PED) and China further highlight its high incidence and apparent heterogeneity, reflecting variations in pathogen profiles, regional factors, and healthcare infrastructure (9–11). This clinical heterogeneity, coupled with the dysregulated host response, makes early prognostication highly challenging, with mortality risk and long-term sequelae such as pediatric acute respiratory distress syndrome (PARDS) difficult to predict at presentation (12, 13). Although scoring systems such as pediatric Sequential Organ Failure Assessment (pSOFA) and Pediatric Logistic Organ Dysfunction score (PELOD-2) are widely used, their reliance on evolving clinical and laboratory data limits their timeliness and precision, particularly for early individualized prognostication (1, 8). Moreover, evolving sepsis definitions—from Systemic Inflammatory Response Syndrome (SIRS)-based International Pediatric Sepsis Consensus Conference (IPSCC) criteria (2005) to the updated Phoenix criteria (2024)—further complicate epidemiological comparisons and undermine consistency in bedside diagnosis and prognostic assessment (14). Collectively, these challenges underscore a critical need for rapid and precise diagnostic tools capable of enabling early risk stratification.

### 2.2 Limitations of traditional diagnostic methods

Conventional diagnostic pathways for pediatric sepsis are slow and insufficiently sensitive. Blood culture, the gold standard for pathogen identification, typically requires 24–72 h and demonstrates limited sensitivity, particularly in children receiving prior antibiotics or with limited sample volumes (15). Consequently, clinicians often initiate empiric broad-spectrum antimicrobial therapy in the absence of definitive microbiological evidence, increasing the risks of antimicrobial resistance and drug toxicity. To complement microbiological data, clinicians rely on a battery of laboratory biomarkers. However, no single biomarker has proven sufficiently sensitive and specific to definitively diagnose sepsis in children (3). Common markers such as C-reactive protein (CRP) and erythrocyte sedimentation rate (ESR) lack specificity, while procalcitonin (PCT), although more discriminative, is still influenced by non-infectious inflammation and shows variable kinetics (3). The literature highlights that despite the identification of over 250 potential sepsis biomarkers, only a few have seen limited integration into clinical decision-making, and none can single-handedly provide an accurate diagnosis (16). As a result, clinicians must rely on nonspecific clinical and laboratory findings, leading to substantial diagnostic uncertainty. This uncertainty contributes to missed or delayed diagnoses, as evidenced in U.S. emergency departments (17), and creates a critical diagnostic gap during the early phase of disease progression.

### 2.3 Time-sensitive nature of intervention

The management of pediatric sepsis is a race against time. The “golden hour” principle emphasizes that early initiation of appropriate therapy is essential, as delays are directly correlated with increased mortality and morbidity (18). It is worth noting that for every hour of delay in administering effective antimicrobial therapy, the risk of mortality increases significantly. However, conventional diagnostic workflows that depend on centralized laboratory testing are fundamentally misaligned with this urgency, resulting in initial empiric management decisions, particularly when distinguishing between viral and bacterial infections (19). Efforts to accelerate recognition, such as electronic health record (EHR)-based screening tools, such as the Sepsis Screening Tool (SST), have shown high sensitivity and specificity; however, delays persist, with approximately 22% of alerts occurring more than one hour after triage (20). This body of evidence points to an undeniable clinical imperative: a paradigm shift from slow, centralized diagnostics to rapid, point-of-care solutions is essential to align diagnostic capability with the time-sensitive therapeutic demands of pediatric sepsis.

### 2.4 Limitations of current POCT technologies

Compared with conventional laboratory testing, point-of-care testing (POCT) still has certain limitations in accuracy and reliability (21). These limitations are not only related to inherent constraints in detection speed and sensitivity, but also largely arise from the complex and dynamic nature of biomarkers. Due to their variability and limited specificity, single biomarkers are often insufficient to provide consistent and reliable diagnostic information (22). However, most current POCT platforms are limited to the detection of one or only a few biomarkers, which significantly affects diagnostic performance (22). At the same time, implementing multiplex detection in POCT remains challenging because of issues such as cross-interference and increased system complexity (23). In addition, some traditional biomarkers used in POCT have inherent drawbacks and may lead to false-positive results (19). POCT methods based on immunoassays, such as lateral flow assays, are also prone to the hook effect, where very high concentrations of the target analyte can produce a reduced signal, resulting in false-negative results and a higher risk of missed diagnosis. Another limitation is the unfavorable kinetic behavior of some commonly used biomarkers (21). For example, CRP typically begins to increase 12–24 h after the onset of inflammation or infection and reaches its peak within 2–3 days, which limits its usefulness for early diagnosis (24). Overall, these challenges highlight the need for the development of next-generation POCT technologies with improved sensitivity, better multiplexing capability, and enhanced performance for early detection. LSPR-based platforms have the potential to address these limitations by offering ultrahigh sensitivity, label-free operation, and multiplexing capability, as detailed in the following sections.

## 3 Fundamentals of LSPR technology

### 3.1 Physical principles of LSPR

Localized surface plasmon resonance (LSPR) arises from the coherent oscillation of conduction electrons at the surface of noble metal nanostructures (e.g., Au, Ag) under resonant optical excitation, constituting the physical basis of highly sensitive, label-free biosensing platforms (7). Unlike propagating surface plasmons in continuous metallic films, LSPR uses discrete nanoparticles with dimensions smaller than the incident light wavelength, resulting in spatially confined plasmon modes, sharp spectral features, and intense near-field enhancement localized at the nanoparticle interface (7, 25).LSPR is intrinsically sensitive to the local dielectric environment. Due to the rapid decay of the evanescent field, refractive index perturbations within a few nanometers of the nanoparticle surface induce measurable shifts in the resonance wavelength. These shifts, typically observed as redshifts, scale with both the refractive index variation and local field intensity, enabling direct optical transduction of molecular binding events at the sensor interface (7).

Key performance descriptors—including refractive index sensitivity (nm/RIU), figure of merit (FoM), and electromagnetic field decay length—are governed by nanoparticle geometry and composition. Advances in nanofabrication enable precise structural control, with anisotropic nanostructures (e.g., nanorods, nanobipyramids, and nanoshells) exhibiting enhanced field confinement and tunable plasmonic responses compared to spherical counterparts, thereby underpinning next-generation LSPR biosensors with superior sensitivity (7, 26).

### 3.2 Mechanistic basis of LSPR biosensing

The translation of LSPR into practical biosensing hinges on the integration of precise surface functionalization and sensitive optical interrogation. Target analytes are selectively captured on plasmonic nanostructures via immobilized biorecognition elements (e.g., antibodies or aptamers), typically anchored through self-assembled monolayers such as mercaptoundecanoic acid (MUA), which enable covalent attachment while minimizing nonspecific adsorption (27, 28). Analyte binding induces local refractive index changes, resulting in a measurable LSPR redshift that supports real-time, label-free quantification. For example, serum interleukin-10 (IL-10) exhibits significantly higher *Δλ*_max_ (the maximum wavelength shift of the LSPR extinction peak) in patients with cancer than in benign controls, underscoring its diagnostic potential (29).

**Figure 1:**
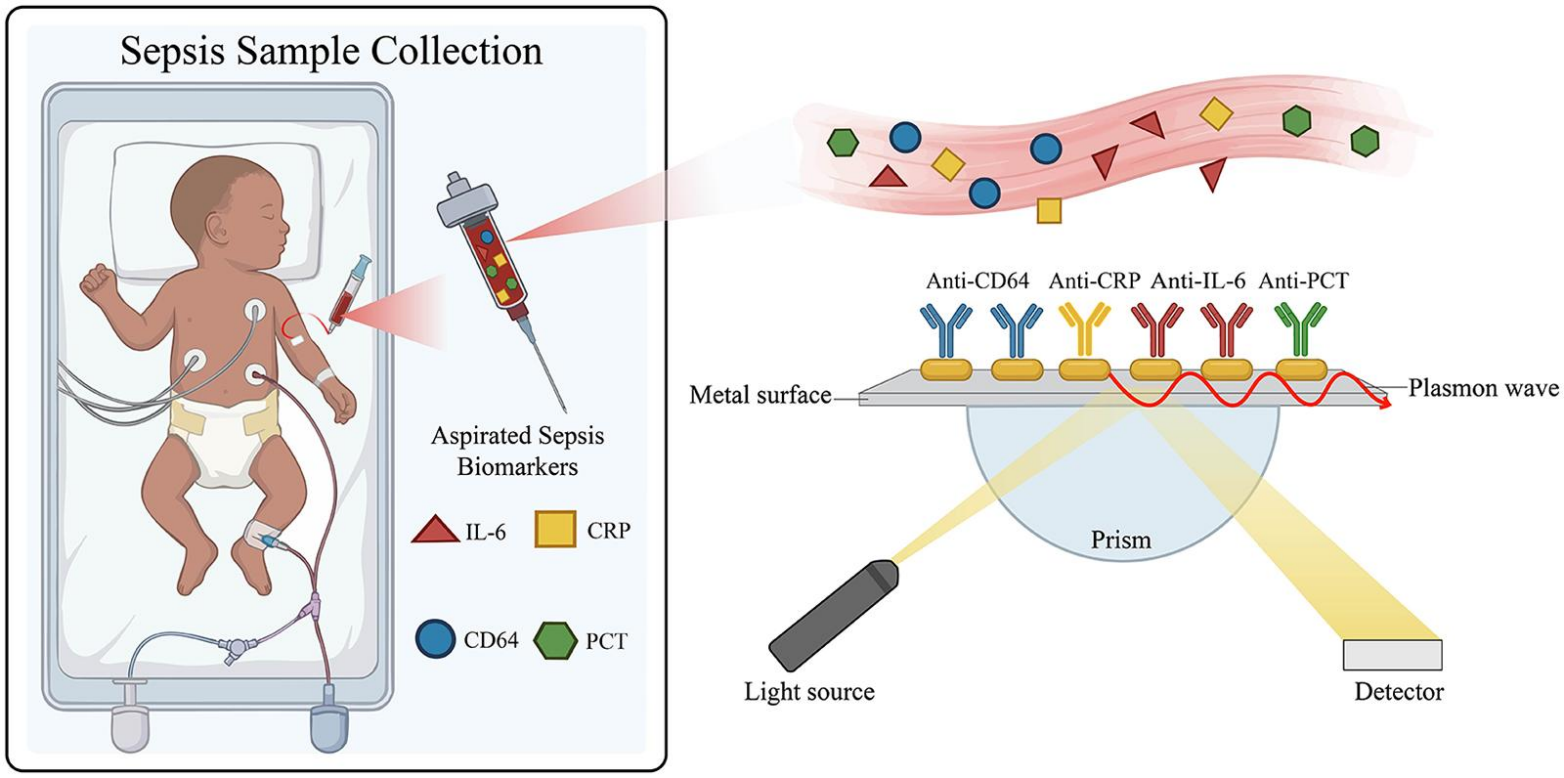
LSPR-based sepsis sample collection using multiplexed biomarker detection. IL-6, interleukin-6; CRP, C-reactive protein; CD64, cluster of differentiation 64; PCT, procalcitonin. Anti-IL-6, anti-interleukin-6 antibody; Anti-CRP, anti-C-reactive protein antibody; Anti-CD64, anti-cluster of differentiation 64 antibody; and Anti-PCT, anti-procalcitonin antibody.

Detection modalities include ensemble measurements, which are operationally simple but limited by inhomogeneous broadening, and single-particle approaches that leverage dark-field scattering for enhanced sensitivity and multiplexing (7, 30). Further performance gains are achieved by digital nanoplasmonometry (DiNM), which refines signal readout via ratiometric spectral analysis, achieving ppb-level detection limits (Figure 1) (31).

### 3.3 Advantages over conventional assays

LSPR-based biosensors exhibit distinct advantages over conventional assays [e.g., enzyme-linked immunosorbent assay (ELISA), radioimmunoassay, and chemiluminescent enzyme immunoassay], making them particularly well-suited for point-of-care applications (32). Foremost, their label-free detection directly transduces molecular binding events into optical signals, eliminating secondary labeling steps, simplifying workflows, reducing assay time, and avoiding label-induced alterations in binding affinity (32). In addition, LSPR sensors achieve exceptional sensitivity, often reaching the femtomolar (fM) regime, as demonstrated by an immuno-silver nanomarble (iSNM) platform for epithelial cell adhesion molecule (EpCAM) detection, enabled by strong electromagnetic field enhancement at nanoparticle surfaces (33).

Beyond sensitivity, LSPR enables rapid, real-time analysis, with detection typically completed within minutes (e.g., IL-10 quantification within 30 min vs. hours for ELISA) (29). Another key advantage lies in miniaturization and portability, as LSPR sensors do not require bulky instrumentation such as plate readers and can be interrogated using simple optical setups or fiber-optic configurations, paving the way for handheld point-of-care devices that require only small sample volumes—an ideal feature for pediatric applications (32, 34). Moreover, multiplexed detection can be achieved using nanoparticle arrays functionalized with distinct biorecognition elements and resonance signatures, allowing simultaneous profiling of multiple biomarkers and providing a more comprehensive assessment of complex diseases such as sepsis (35).

## 4 LSPR-based POCT systems for sepsis

### 4.1 Platform performance and biomarker panel

Recent technological advances in SPR and LSPR have made multiplexed detection feasible with exceptional analytical performance (Table 1). The clinical utility of LSPR-based point-of-care (POC) systems for sepsis hinges on a multiplexed approach that captures the complex interplay of inflammatory, anti-inflammatory, and immunosuppressive pathways. A single biomarker is insufficient; thus, the most promising strategy involves simultaneous measurement of key targets, including pro-inflammatory cytokines [e.g., interleukin-6 (IL-6), tumor necrosis factor-alpha (TNF-α)], acute-phase proteins (e.g., CRP, PCT), and novel markers like pancreatic stone protein (PSP) or neutrophil cluster of differentiation 64 (CD64) (3, 29, 36–38). Emerging proteomic and machine learning approaches are further refining these panels to enable precise diagnosis, etiological classification, and prognostic risk stratification (12, 39, 40).

**Table 1:** Advanced biosensing platforms for sepsis biomarker detection.

Platform	Target biomarkers	Limit of detection (LOD)	Detection time	Key performance features	Reference
SPR Imaging Array	IL-6	1.1 pg/mL	–	High sensitivity	(41)
LSPR (Au nanorods with peptide aptamer)	IL-6	4.6 pg/mL (linear up to 1 × 10^6^ pg/mL)	35 min	Higher sensitivity than antibodies; wide linear range (5–10^6^ pg/mL); strong ELISA correlation	(41)
cDiNM	IL-6	<19.2 fg/mL	45 min	Ultra-high sensitivity, 96% recovery	(42)
MetaSPR Biosensor	PCT, CRP, SAA	39 pg/mL	Real-time	excellent correlation with latex turbidimetric method (R^2^ = 0.899)	(43)
3D Nanopillar SERS	CD123, PD-L1, HLA-DR, Chitotriosidase	4–6 fM	60 min	High reproducibility (RSD = 1.79%); 95% classification accuracy with ML	(44)
SERS-DL	Multiple blood biomarkers	–	–	Very high accuracy: 99.67% (sepsis detection), 98.84% (pathogen classification)	(45)
				Strong generalization: 98.28% accuracy on external cohort	
Magnetic Phage-Based SERS	CRP, PCT, sTREM-1	pM range	60 min	Multiplex (triplex) detection capability. High sensitivity with pM-level LOD. Good specificity with minimal cross-reactivity.	(46)
Dual-mode Colorimetric/SERS Sensor	Staphylococcus aureus (MNase)	38 CFU/mL (colorimetric);	90 min	Good reproducibility (RSD = 5.42%); high specificity; AUC = 1.00; therapy monitoring capability	(47)

For the core biomarker IL-6, SPR imaging arrays achieved limits of detection (LODs) as low as 1.1 pg/mL, while LSPR sensors using gold nanorods achieved LODs of 4.6 pg/mL with a wide linear range up to 1 × 10^6^ pg/mL (41). A novel colorimetric digital nanoplasmonometry (cDiNM) method pushed LODs to sub-20 fg/mL (0.8 fM) in plasma, with a recovery rate of 96% (42). These assays are rapid, with total detection times of 30–45 min, significantly faster than traditional ELISA. Furthermore, a metasurface plasmon resonance (MetaSPR) biosensor demonstrated high-throughput, real-time quantification of PCT, CRP, and serum amyloid A (SAA) in 80 clinical plasma samples, achieving an LOD of 39 pg/mL and excellent correlation (R^2^ = 0.899) with reference methods (43).

Notably, the strong electromagnetic field enhancement underlying LSPR also forms the basis of surface-enhanced Raman scattering (SERS), which has been widely adopted for sepsis biomarker detection. A 3D nanopillar-based SERS biosensor simultaneously detected four immune-related proteins [CD123, programmed death-ligand 1 (PD-L1), human leukocyte antigen-DR (HLA-DR), and chitotriosidase] with LODs of 4–6 fM and high consistency (relative standard deviation, RSD = 1.79%) (44). When combined with machine learning, this platform achieved 95.0% accuracy in classifying patients into healthy, infected, septic, and septic shock groups, demonstrating utility for both diagnosis and severity staging (44). In a large-scale study of 653 blood samples, a SERS-deep learning (SERS-DL) workflow using a neural network classifier achieved 99.67% accuracy for binary sepsis identification and 98.84% accuracy for classifying six pathogen types, with strong generalizability validated in an external cohort (98.28%) (45). Another SERS-based platform using phage-displayed magnetic templates enabled simultaneous detection of CRP, PCT, and soluble triggering receptor expressed on myeloid cells-1 (sTREM-1) with LODs in the pM range and achieved clinical sensitivity and specificity exceeding 90% (area under the curve, AUC > 0.95) (46).

A recently developed colorimetric/SERS dual-mode sensor exemplifies the integration of these principles. Utilizing an Au@AgPt nanozyme array and a micrococcal nuclease (MNase)-specific cascade strategy, this platform detected Staphylococcus aureus with LODs of 38 colony-forming units (CFU)/mL (colorimetric) and 6 CFU/mL (SERS) (47). It demonstrated excellent repeatability (RSD = 5.42%), high specificity against interfering bacteria, and perfect discriminatory ability (AUC = 1.00) in a clinical cohort of 10 patients. Importantly, it also enabled monitoring of treatment response, showing a significant decrease in MNase levels after antibiotic therapy (47).

Collectively, these SPR, LSPR, and SERS-based platforms, many incorporating advanced nanostructures, machine learning, and dual-signal outputs, represent a paradigm shift. They provide rapid, culture-free, and highly accurate sepsis diagnosis and pathogen identification, establishing a robust foundation for the next generation of point-of-care testing. Despite these impressive analytical performances, the clinical translation of these platforms requires careful evaluation of their integration into real-world workflows, cost-effectiveness, and regulatory pathways, as discussed below.

### 4.2 Clinical heterogeneity and emerging POCT technologies

The marked clinical heterogeneity of pediatric sepsis presents a substantial challenge to diagnostic modalities. Across ages, infecting pathogens, and immune states, the concentration–time trajectories of biomarkers vary markedly. For instance, the magnitude of CRP elevation differs between preterm and term neonates; the peak timing of IL-6 varies between Gram-negative bacterial and viral infections; and in immunosuppressed children, classical biomarker surges may be blunted or entirely absent throughout the disease course (48, 49). Consequently, conventional diagnostic strategies that rely on a single time point and uniform cutoff thresholds may miss early diagnoses in certain subgroups while yielding false positives in others (48, 49). This intrinsic limitation has spurred rapid development of novel point-of-care testing (POCT) technologies designed to capture the multidimensional complexity of the disease process.

With respect to pathogen identification and host biomarker detection, multiplex polymerase chain reaction (PCR) enables direct detection of up to twenty pathogens with high sensitivity within a total turnaround time of 2–4 h. However, its ability to detect low-level infections or polymicrobial co-infections remains limited (50). Electrochemical biosensors offer portability and low cost, achieving femtogram-per-milliliter (fg/mL) sensitivity through direct protein detection and rapid assay times of 3–5 min. Some platforms support multiplexing of up to seven analytes; however, their long-term stability in complex biological matrices remains a key limitation (51–53). Microfluidic devices provide high integration and minimal sample requirements, but their multiplexing capability is limited to 2–3 targets, with assay times ranging from roughly 10 to 90 min (22, 54). Paper-based devices offer the greatest simplicity, with assay times of 10–15 min and substantial utility in resource-limited settings; however, their analytical sensitivity and quantitative performance remain constrained, and outputs are generally qualitative or semi-quantitative (55, 56).

Against this technological landscape, localized surface plasmon resonance (LSPR) furnishes a distinctive complementary solution. Unlike many electrochemical sensors that may suffer from matrix interference and limited long-term stability, LSPR maintains fg/mL-level sensitivity directly in plasma with a wide dynamic range and no spectral crosstalk, making it particularly suited for multiplexed host-response profiling (41–43, 46). Accordingly, coupling LSPR with microfluidic systems enables sample preprocessing, fluid handling, and optical readout to be unified on a single platform, yielding fully automated “sample-in, result-out” POCT. When integrated with multiplex PCR, LSPR may further support stratified diagnostic workflows by providing complementary information: PCR enables pathogen identification and antimicrobial resistance profiling, whereas LSPR provides quantitative reflection of the host immune–inflammatory status. When such high-dimensional POCT data are integrated with clinical variables through machine-learning models, the combined approach may further dissect the heterogeneity of pediatric sepsis, moving beyond single-biomarker cutoff logic. Emerging studies indicate that machine-learning models integrating multimodal clinical data can predict sepsis up to 24 h before physician suspicion, achieving an AUC of 0.82 (57).

Notably, a shared limitation across these technologies is that validation of diagnostic performance remains predominantly based on single time-point measurements. In recent years, intensive care research has increasingly highlighted the value of longitudinal trajectory analysis from methodological and clinical perspectives. Methodologically, Xia et al. (58) systematically compared six longitudinal trajectory modeling approaches and demonstrated that conventional static phenotyping may obscure dynamic variations in disease evolution. Moreover, multiple real-world studies across diverse domains—including Sequential Organ Failure Assessment (SOFA) scores, hemodynamics, protein biomarkers, and metabolic indicators—have consistently shown that trajectory-based stratification conveys substantially richer clinical information than single time-point measurements, with mortality risk differing by several folds across trajectory groups, underscoring its utility for both diagnosis and risk stratification (59–61). In pediatric populations, Yehya et al. (62) and Wang et al. (63), employing temperature trajectories in 191 children and glucose trajectories in 1,178 children respectively, further demonstrated the value of longitudinal data–driven phenotyping in identifying clinical subtypes, guiding prognostic stratification, and enabling individualized interventions. Collectively, these findings indicate that longitudinal trajectory analysis captures disease evolution patterns inaccessible to snapshot measurements. Extending this framework to rapid diagnostic platforms such as LSPR or electrochemical sensors implies that continuous biomarker monitoring, coupled with trajectory-based models, may enable more robust interpretation of dynamic biological signals. This approach holds the potential to sustain high diagnostic performance across heterogeneous patient subgroups while reducing misdiagnosis and missed diagnoses associated with reliance on single time-point cutoff values.

## 5 Clinical impact and implementation

### 5.1 Health economics and cost-effectiveness analysis

The clinical translation of emerging diagnostic technologies rests not only on analytical performance but also on economic feasibility. Although the per-test cost of LSPR-POCT may exceed that of conventional laboratory assays, pathway-based health economic evaluations suggest that these incremental costs can be offset by downstream savings across the care continuum. When configured with a panel of host-response biomarkers, LSPR-POCT has the potential to rapidly discriminate between bacterial and non-bacterial infections, shortening the duration of empiric broad-spectrum antibiotic therapy, reducing drug-related expenditures, and mitigating the long-term economic burden associated with antimicrobial resistance and related complications (19). Its capacity to rapidly identify sepsis and stratify disease severity can accelerate the initiation of sepsis bundles and other time-critical interventions (46). Early risk stratification further allows precise identification of high-risk pediatric patients requiring escalation of care, while avoiding unnecessary intensive care unit (ICU) admissions in low-risk cases, thereby improving outcomes and optimizing healthcare resource allocation (64).

Moreover, the extremely low sample-volume requirement represents a distinct pediatric advantage. For example, only 50 µL of blood is needed for a 5 kg infant with an LSPR-based assay, vs. 2–3 mL for conventional methods, potentially reducing hidden costs associated with iatrogenic anemia, transfusion requirements, and prolonged hospitalization in neonates and infants. Although dedicated health economic evaluations of LSPR-POCT are still sparse, evidence from other POCT strategies provides useful benchmarks. Modeling-based analyses suggest that a neonatal sepsis POCT strategy in hospital settings could reduce mortality by 19% and decrease healthcare costs by 17%–43%, achieving cost neutrality at a per-test cost not exceeding $21 USD; in community settings, mortality reductions reach up to 70%, with cost savings of 48%–81% (65). This study underscores that even moderately accurate POCT systems can simultaneously improve clinical outcomes and reduce costs by expediting diagnosis and avoiding unnecessary hospitalization. Importantly, its analytical framework is directly applicable to LSPR-POCT.

Taken together, although the precise cost-effectiveness profile of LSPR-POCT awaits validation in prospective studies, the conceptual analysis indicates that its advantages in rapid diagnosis, optimized antimicrobial use, and improved resource allocation—coupled with the low sample-volume requirement in pediatric populations—are more likely to reduce, rather than inflate, the overall cost of sepsis management. Future clinical investigations should embed structured health-economic endpoints from the outset to enable robust cost-effectiveness evaluation.

### 5.2 Bias control in diagnostic performance and integration into clinical workflow

Translating LSPR-POCT from laboratory validation to real-world clinical deployment is beset by substantial biases that can arise at multiple stages of the diagnostic cascade. Even when analytical performance is robust under controlled conditions, accuracy may deteriorate in routine clinical practice, and the absence of standardized protocols and rigorous quality-control measures may further amplify these limitations. Systematic identification and mitigation of these bias sources are therefore essential to sustaining clinical utility in practice.

At the level of evidence generation, current diagnostic-accuracy studies of LSPR platforms are predominantly small-scale, single-center, case–control designs. When evaluated with the Quality Assessment of Diagnostic Accuracy Studies-2 (QUADAS-2) framework, several recurrent biases are observed, including spectrum bias in patient selection (where healthy volunteers rather than consecutively enrolled suspected cases serve as controls), review bias in index-test interpretation (where reference-standard results are known at interpretation or diagnostic thresholds are optimised *post hoc*), incorporation bias in the reference standard (notably owing to the limited sensitivity of blood culture and the presence of culture-negative sepsis), and flow-and-timing bias (where biomarker measurements are performed later in the disease course rather than within the intended early diagnostic window). Collectively, these methodological limitations may inflate reported sensitivity and specificity (66).

Beyond these concerns, even high-quality studies may not fully reflect real-world performance, as diagnostic accuracy is expected to degrade when LSPR-POCT moves from controlled laboratory environments into clinical settings. Contributing factors include the markedly more complex composition of clinical samples (e.g., hemolysis, lipemia, nonspecific protein interference) relative to spiked laboratory specimens; the shift of operators from trained researchers to emergency physicians or bedside nurses; environmental influences such as temperature and humidity fluctuations on nanomaterial stability; and the expansion of the patient spectrum from selectively enrolled cohorts to unselected, consecutive suspected cases. Moreover, in resource-limited settings, extreme temperature and humidity conditions, unstable power supplies, and challenges in cold-chain reagent transport may further exacerbate performance degradation. Together, these factors constitute external validity bias that can substantially affect clinical performance.

Given that LSPR-POCT is intended for high-stakes decision points—such as triage discrimination of bacterial vs. non-bacterial infection, serial therapeutic monitoring, and pre-discharge clearance verification—any degradation in accuracy could directly lead to inappropriate antibiotic use or unsafe disposition. A structured quality-assurance framework is therefore essential to preserve performance across these clinical contexts. An ideal system would operate as a fully automated, closed “sample-in, result-out” platform, whereby clinicians perform only minimal sample loading and results are directly integrated into electronic medical records and linked to clinical decision-support systems. In this configuration, automation substantially simplifies workflow, shifting training requirements from procedural proficiency to standardized sample handling and interpretation of multiplex quantitative outputs within the clinical context. From a quality-control perspective, built-in automatic calibration and daily internal quality checks are essential to ensure analytical stability, complemented by external proficiency testing and inter-device comparisons to establish a robust dual-layer quality-assurance framework.

### 5.3 Causal inference and target trial emulation

While the quality-assurance framework ensures that LSPR-POCT operates reliably in clinical settings, demonstrating that this reliable operation translates into improved patient outcomes—such as lower mortality or shorter hospitalization—requires direct causal evidence. The randomized controlled trial (RCT) remains the gold standard for establishing causality; however, in pediatric sepsis, ethical considerations and substantial implementation costs frequently constrain such trials.

Target trial emulation (TTE) offers a principled alternative by leveraging observational data to approximate the design of a hypothetical RCT, thereby enabling estimation of causal effects (67). In the emergency setting of children with suspected sepsis, the key elements of an idealized target trial can be precisely defined and emulated using electronic health record data to evaluate the causal impact of an LSPR-POCT–guided diagnostic strategy vs. conventional blood-culture–based pathways on outcomes such as mortality, hospital length of stay, antibiotic use, and time to appropriate antimicrobial therapy.

A growing body of evidence demonstrates the utility of target trial emulation in sepsis-related interventions. For example, using a TTE framework, early ICU transfer in septic shock was estimated to reduce mortality risk by 52%, and early initiation of adjunctive vasopressin therapy was estimated to reduce 30-day ICU mortality by approximately 5% (68, 69). These studies illustrate how TTE can generate causal estimates in contexts where randomization is infeasible, suggesting that the same framework could be applied to evaluate the causal impact of an LSPR-POCT–guided diagnostic strategy. Nonetheless, TTE rests on assumptions—exchangeability, positivity, and correct model specification—that require careful attention. Sensitivity analyses and transparent reporting are essential to mitigate residual bias and to interpret causal estimates credibly.

When its underlying assumptions are reasonably satisfied, TTE can complement RCTs by providing timely causal estimates with strong external validity, informing trial design, resource allocation, and policy decisions, while guiding the iterative refinement of LSPR-POCT–driven diagnostic workflows.

### 5.4 Regulatory pathways and market authorization requirements

Despite the notable clinical potential of LSPR-POCT, translation from laboratory prototype to commercial deployment requires rigorous regulatory clearance to demonstrate safety, effectiveness, and robust quality control. In the United States, LSPR-POCT would fall under the purview of the Food and Drug Administration (FDA). Given its reliance on nanomaterials and its application in high-risk pediatric sepsis scenarios, prospective multicenter clinical trial data would almost certainly be required to support a favorable decision. Given its reliance on nanomaterials—which introduce concerns about long-term biocompatibility and batch-to-batch consistency—and its intended use in high-risk pediatric sepsis scenarios, regulatory authorities would almost certainly expect prospective multicenter clinical trial data to demonstrate both safety and effectiveness in the target population. In the European Union, CE marking would be necessary, and depending on the device's risk classification, conformity assessment by a Notified Body would be anticipated under the Medical Devices Regulation (MDR).

Regardless of jurisdiction, regulatory approval typically hinges on three core evidentiary pillars. First, analytical validity, including verification of the limit of detection, precision, and batch-to-batch consistency of nanostructured materials. Second, clinical validity, established through prospective studies in the intended patient population to determine diagnostic sensitivity and specificity. Third, clinical utility, demonstrating that device implementation yields measurable improvements in patient management and clinical outcomes.

LSPR-POCT presents several distinctive regulatory challenges. Notably, its analytical targets are host-response biomarkers rather than pathogen-derived signals. Because such biomarkers can be framed as aiding in diagnosis, prognosis, or risk stratification, the manufacturer's intended use statement will directly shape the device's regulatory classification and the corresponding evidence requirements. In addition, the stability and reproducibility of nanomaterials lack universally harmonized international standards, complicating the demonstration of analytical validity. These uncertainties can be best addressed through a proactive regulatory strategy, as discussed below (70).

Beyond the core evidentiary pillars, a comprehensive quality-management framework must extend across the product lifecycle. At the device-software interface, regulatory strategies should address software as a medical device (SaMD) considerations, cybersecurity resilience, and the quality-management system in accordance with ISO 13485 and relevant product standards. In parallel, manufacturing controls for nanomaterials (biocompatibility, residuals, and nanoparticle characterization) should align with ISO 10993 and related guidelines, with explicit risk management documented per ISO 14971. Post-market surveillance plans, adverse-event reporting, and periodic re-evaluation of performance against real-world data will be essential components of sustained regulatory compliance.

Engagement with regulatory authorities should be proactive and iterative. Early pre-submission meetings (FDA) or scientific advice procedures (EU) can clarify device classification, expected study designs, and acceptable clinical endpoints. A carefully staged regulatory plan—encompassing analytical validation, pivotal clinical studies, real-world evidence generation, and a clearly defined post-market plan—will best position LSPR-POCT for successful authorization while mitigating the regulatory uncertainties described above.

In sum, while the road to regulatory approval for LSPR-POCT is complex and highly contingent on jurisdiction, a disciplined, evidence-driven strategy—spanning rigorous analytical and clinical validation, clearly demonstrated clinical utility, and a comprehensive quality-management system that encompasses software, nanomaterials, and post-market obligations—will be decisive. Collaboration with regulators, standard-setting bodies, and clinical stakeholders from the earliest developmental stage can help harmonize expectations, accelerate access, and ensure that the technology delivers on its promise to improve pediatric sepsis care (70).

## 6 Conclusions

This review highlights the emerging potential of localized surface plasmon resonance (LSPR)-based point-of-care testing (POCT) for pediatric sepsis, a condition that demands rapid and accurate diagnosis due to its high mortality and critically time-sensitive therapeutic window. Conventional assays remain inadequate, constrained by slow turnaround and limited specificity, thereby leaving a critical diagnostic gap in early-stage disease. LSPR, with its foundation in the highly sensitive optical transduction of molecular binding events on noble metal nanostructures, offers a compelling solution. Its intrinsic features—label-free detection, high sensitivity, multiplexing capability, and compatibility with miniaturized platforms—align perfectly with the requirements of an ideal POCT device for a complex syndrome such as sepsis.

Translating LSPR-POCT into clinical practice requires fully integrated platforms combining functionalized nanostructures, microfluidics, and compact optical readouts for rapid bedside biomarker profiling. The selection of an optimal panel of inflammatory, infectious, and immune response biomarkers is crucial for diagnostic accuracy and is an area of active research. Despite promising proof-of-concept studies, it is important to acknowledge that most current evidence comes from small-sample or single-center studies. Large-scale, prospective, multicenter clinical validation remains a key challenge, along with regulatory, economic, and clinical workflow integration barriers. Priority should be given to prospective multicenter trials enrolling diverse pediatric populations to establish robust cutoff values and demonstrate clear clinical utility.

Overall, LSPR-based POCT holds transformative potential to shift pediatric sepsis care toward rapid, data-driven decision-making, enabling earlier intervention and improved clinical outcomes. Realizing this promise will depend on coordinated interdisciplinary efforts to bridge the gap from innovation to widespread clinical adoption.
